# An Alternating Magnetic Field-Controlled Drug Delivery System Based on 4,4′-Azobis (4-cyanovaleric Acid)-Functioned Fe_3_O_4_@Chitosan Nanoparticles

**DOI:** 10.3390/bioengineering10020129

**Published:** 2023-01-18

**Authors:** Wang Yin, Randy Bachelard Nziengui Raby, Yuankai Li, Zuojun Li, Mengqing Sun, Zhi Huang

**Affiliations:** 1 Institute of Biomedical Engineering, School of Basic Medical Sciences, Central South University, Changsha 410017, China; 2 Department of Pharmacy, the Third Xiangya Hospital, Central South University, Changsha 410013, China

**Keywords:** superparamagnetic nanoparticle, chitosan, alternating magnetic field, magneto–thermal, drug delivery system, thermosensitive molecule, controlled release

## Abstract

Herein, we designed chitosan–coated Fe_3_O_4_ nanocomposites for the control release of drugs by an alternating magnetic field (AMF). The chitosan-coated Fe_3_O_4_ nanoparticles (Fe_3_O_4_@CS) were prepared by a alkaline co-precipitation method, and then, the model drug toluidine blue (TB) was covalently grafted onto the surface of the nanocomposite by a two-step amide reaction with the thermosensitive molecule 4,4′-azobis (4-cyanovaleric acid) (ACVA) as the linker group. The prepared nanocomposites were superparamagnetic and showed high magnetization saturation (about 54.0 emu g^−1^). In vitro hydrothermal release studies showed that most parts of the TB would be effectively enclosed within the nanocarriers at lower ambient temperatures (23 or 37 °C) due to the molecular bonding of ACVA. The results of kinetic fitting of hydrothermal release data showed that TB released from nanoparticles followed first-order kinetics (R^2^ > 0.99) and the Korsemeyer–Peppas model (R^2^ > 0.99, n < 0.5). Most importantly, a single magnetron release experiment demonstrated an approximately linear relationship between the cumulative release of the drug and the duration of action of AMF (R^2^ = 0.9712). Moreover, the increase in the cumulative release of the drug can be controlled by controlling the switch of the AMF generation device. Therefore, the ACVA-modified Fe_3_O_4_@CS nanocarrier designed in this study is a promising model for drug delivery that enables the control of drug release dose by AMF.

## 1. Introduction

In recent decades, drug delivery system (DDS) based nanomaterials have gained numerous anticipated achievements, which have increasingly become an essential strategy for diagnosis and disease therapy in the biomedical field [1]. However, the necessity of delivering precise doses to specific sites of diseases at defined times remains a challenge [2]. Stimulus-responsive nanocarriers serve as an intelligent and effective drug delivery platform that can reduce the side effects of the drug and improve the therapeutic efficacy [3]. These stimuli include endogenous (pH, enzymes, and redox) and exogenous (temperature, ultrasound, light, and magnetic fields) [4]. In contrast, the responsiveness of nanocarriers can be designed by the properties of the material itself or by modifying appropriate release triggers [5,6]. Among them, magnetic nanocarrier systems based on external alternating magnetic field (AMF) response have the following particular advantages: (a) no penetration depth restriction by magnetic fields to trigger drug release in the deep animal body without scattering or absorption by tissues, such as light, and (b) less limitation on the type of tissue treated than ultrasound methods strongly attenuated by air and bone structure [7].

Due to their unique magnetic separation properties, good biocompatibility, and low toxicity, Fe_3_O_4_ nanoparticles have emerged as effective candidates for drug delivery systems [8,9,10]. Additionally, when exposed to an external alternating magnetic field (AMF), Fe_3_O_4_ nanoparticles exhibit excellent magneto-thermal conversion properties due to Néel and Brownian relaxation [11,12]. This distinctive stimulus responsiveness of Fe_3_O_4_ nanoparticles facilitates the release of loaded drugs and can work synergistically with thermal therapy to enhance the therapeutic effect. Nevertheless, the bare Fe_3_O_4_ nanoparticles have some limitations as a DDS, including difficulty controlling drug release and low loading capacity [13]. To construct a magnetic drug delivery system based on Fe_3_O_4_ nanoparticles with high magnetic saturation, biocompatibility, and interactive function, it is necessary to precoat the surface with a coating that makes it stable, biodegradable, and non-toxic in the physiological medium [14].

Many multifunctional nanocarriers composed of functional inorganic nanomaterials and protective organic matrices have promising applications in bioimaging, diagnosis, and therapy [15]. In addition to creating more hydrophilic nanostructures, the polymer coating also provides functional groups to the surface to inhibit aggregation, enhance stability and immobilize the drug [16]. With ample amino and hydroxyl functional groups, chitosan (CS) is a renewable, positively charged natural biopolymer [17,18,19]. Thus, CS-coating not only protects and stabilizes magnetic nanoparticles but also allows the nanoparticles to be functionalized with specific components.

**Scheme 1:**
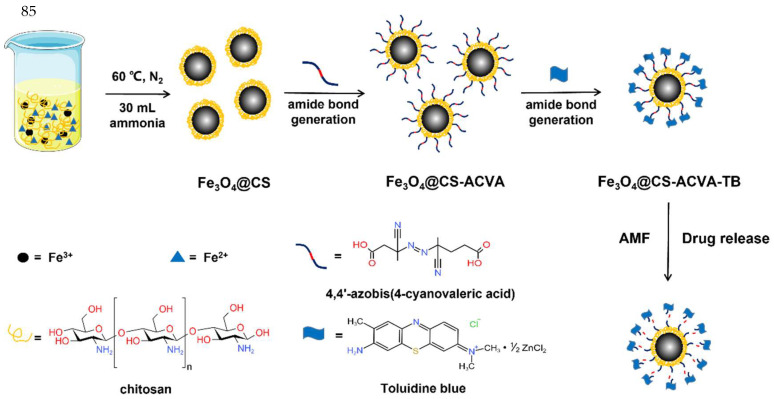
Synthesis schematic of azo-functionalized chitosan-coated Fe_3_O_4_ nanoparticles and triggered release under an AMF.

**Scheme 2:**
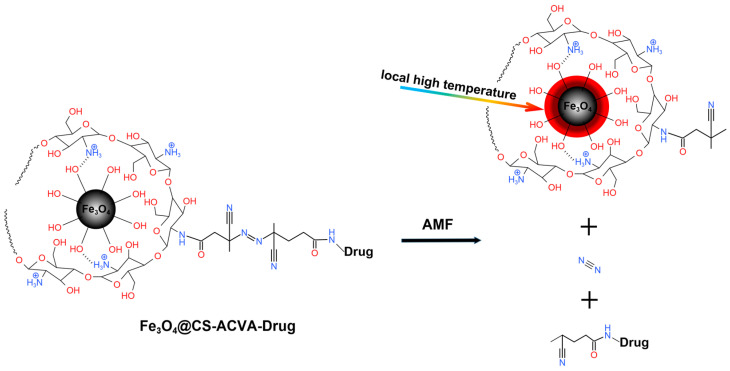
The structure schematic of the drug released from Fe_3_O_4_@CS nanoparticles under an AMF.

Here, we designed an Fe_3_O_4_@CS nanoparticle that achieves time-dependent control of drug release by adjusting the AMF exposure time. CS-modified Fe_3_O_4_ as a drug transport carrier was firstly prepared by alkaline co-precipitation, followed by a two-step amide reaction to covalently graft drug molecular onto the Fe_3_O_4_-CS surface by using a heat-sensitive molecule ACVA as a linker group. To study drug release, toluidine blue (TB), a thiazide variant dye carrying a reactive amino group, was used as a model drug. Due to the high molar absorption coefficient, good thermal stability, and water solubility, TB has been widely used in biomedical fields [20]. Magnetic chitosan nanoparticles loaded with TB were synthesized (as in Scheme 1). Afterward, the physical and chemical properties of the nanoparticles were characterized by Fourier transform infrared spectrophotometer (FT-IR), X-ray diffraction (XRD), transmission electron microscopy (TEM), thermogravimetry (TGA), dynamic light scanning (DLS) and vibrating sample magnetometer (VSM). Then, the magneto-thermal conversion properties and specific absorbed power of Fe_3_O_4_@CS nanoparticles were investigated by applying AMF. In vitro release behavior of magnetic chitosan nanoparticles loaded with TB at different water bath temperatures as well as different AMF action conditions (as in Scheme 2) was investigated with emphasis, and the release kinetics were analyzed and discussed.

## 2. Materials and Methods

### 2.1. Materials

Multiple reagents such as ferric chloride hexahydrate, ferrous sulfate heptahydrate, ammonia, hydrochloric acid, sodium hydroxide, chitosan (CS), toluidine blue (TB), 4-(4,6-dimethoxytriazin-2-yl)-4-methyl morpholine hydrochloride (DMTMM), and 4,4′-azobis-(4-cyanovalerate acid) (ACVA) were purchased from Macklin Biochemical Co., Ltd. (Shanghai, China). The acetic acid and anhydrous ethanol were purchased from Sinopharm Chemical Reagent company. All chemicals used were of analytical grade and were used without further purification.

### 2.2. Synthesis of Fe_3_O_4_@CS Magnetic Nanoparticle

The Fe_3_O_4_@CS nanoparticles were synthesized using modified co-precipitation method [21]. First, a total of 0.01 mol of FeCl_3_·6H_2_O (2.203 g) and 0.005 mol of FeSO_4_·7H_2_O (1.89 g) were dissolved in 25 mL of deionized water (named A). Subsequently, 0.5 mL of acetic acid and 0.5 g of CS were dissolved in 50 mL of deionized water (named B). Then, in a 250 mL three-neck flask equipped with a mechanical stirring bar, 25 mL of A and 25 mL of B were mixed vigorously stirring at a rate of 150 r/min under the flow of nitrogen for 10 min. When the mixture was heated to 60 °C, 20 mL ammonia was added and stirred for another 1 h under N_2_ at 60 °C. The product was washed with deionized water and anhydrous ethanol. The final product was freeze-dried and named as Fe_3_O_4_@CS-1. Fe_3_O_4_@CS-2, Fe_3_O_4_@CS-3, Fe_3_O_4_@CS-4, and Fe_3_O_4_@CS-5 magnetic nanoparticles with CS contents of 2, 3, 4, and 5 wt.% were also prepared, respectively, by altering the CS weight ratio in solution B described above. All the samples obtained were stored at 4 °C for further investigations.

### 2.3. Synthesis of Fe_3_O_4_@CS-ACVA-TB Magnetic Nanoparticles

Fe_3_O_4_@CS-ACVA nanoparticles were synthesized as follows. Firstly, 2.7 mmol of ACVA (756.8 mg) was dissolved in 1% (*w*/*w*) NaOH solution, and then, the pH was adjusted to 7.2 through NaOH/HCl (1 M). Secondly, 2.7 mmol of DMTMM (747.1 mg) was added to activate the carboxylic acid of ACVA for 20 min at 4 °C. Thirdly, 0.9 mmol of Fe_3_O_4_@CS-1 was added, and then, the pH was adjusted to 7.2 and stirred for 5 days at 4 °C ice bath. Finally, the product was washed for several times with deionized water and named as Fe_3_O_4_@CS-ACVA.

Subsequently, Fe_3_O_4_@CS-ACVA was dispersed in 100 mL deionized water, and then, 0.9 mmol of DMTMM (249.048 mg) was added to activate the carboxylic acid of Fe_3_O_4_@CS-ACVA for 20 min at 4 °C. After that, 0.9 mmol of TB (336.6 mg) was added to the activated Fe_3_O_4_@CS-ACVA in deionized water, and then, the pH was adjusted to 7.2 through NaOH/HCl (1 M) and stirred for 5 days at 4 °C. Finally, the product was washed several times with deionized water and freeze-dried, named as Fe_3_O_4_@CS-ACVA-TB.

### 2.4. Characterization of the Magnetic Nanoparticles

The surface group information of Fe_3_O_4_@CS-1, Fe_3_O_4_@CS-ACVA, and Fe_3_O_4_@CS-ACVA-TB was analyzed by Fourier transform infrared spectrophotometer (FT-IR, Nicolet iS-5, Thermos Fisher Scientific, Waltham, MA, USA) in the range of 4000–400 cm^−1^ using 150 scans. The inorganic crystal composition information of Fe_3_O_4_@CS-1 and Fe_3_O_4_@CS-ACVA-TB magnetic nanoparticles was determined by X-ray diffraction (XRD, Bruker-D8, Germany, Empyrean with Cu-Kα radiation, *λ* = 1.5418 Å), and the XRD patterns were scanned from 2θ = 20°–80°. Furthermore, the organic–inorganic contents from Fe_3_O_4_@CS-1, Fe_3_O_4_@CS-ACVA, and Fe_3_O_4_@CS-ACVA-TB were characterized through thermogravimetric analysis (TGA, STD-650, TA Instruments, USA) in the extent of 25 to 800 °C under N_2_ protect. The morphology of Fe_3_O_4_@CS-ACVA-TB magnetic nanoparticles was investigated by transmission electron microscopy (TEM, FEI, Talos f200s, Thermos Fisher Scientific, Waltham, MA, USA). Moreover, the size of Fe_3_O_4_@CS-1 and Fe_3_O_4_@CS-ACVA-TB nanoparticles distribution and hydrodynamic diameter were determined by dynamic light scattering (DLS, BeNano 90, Dandong, China). DLS measurements were performed using 50 mW laser excitation at 671 nm and at a back scattering angle of 173° at 25 °C. All samples (diluted with deionized water to 1 μg/mL) were tested three times. Magnetic measurements were carried out at 300 K and ranged from −20,000 to about +20,000 Oe, using a vibrating sample magnetometer (VSM, Lake Shore 7404, NY, USA). The concentration of released drug was studied using a UV–visible spectrophotometer (UV-1800CS, Macylab Instrument Inc., Shanghai, China), equipped with a thermostatic cell compartment at room temperature.

### 2.5. Magneto-thermal Properties Analysis of Fe_3_O_4_@CS Nanoparticles

The measurement of magnetic heating characteristics of the Fe_3_O_4_@CS nanoparticles was performed by using a homemade magnetic hyperthermia device. To evaluate the influence of AMF on the magneto-thermal properties of MNPs, Fe_3_O_4_@CS-1 suspension (3 mg/mL) was exposed to AMF with intensities of 2.64 × 10^8^, 4.23 × 10^8^, 5.82 × 10^8^ and 7.40 × 10^8^ Am^−1^s^−1^ for 5 min. The cyclic magneto-thermal properties under AMF with intensities of 5.82 × 10^8^ Am^−1^s^−1^ for 10 min were also determined at the same time. Additionally, the effect of the concentration of Fe_3_O_4_@CS nanoparticles and the CS content were evaluated. The former was explored by placing the Fe_3_O_4_@CS-1 suspensions with concentrations of 0.5, 1, 3, 6, and 9 mg/mL under AMF with the intensity of 5.82 × 10^8^ Am^−1^s^−1^ for 5 min. Yet, the latter was determined by placing 5 mg/mL of Fe_3_O_4_@CS-1, Fe_3_O_4_@CS-2, Fe_3_O_4_@CS-3, Fe_3_O_4_@CS-4, and Fe_3_O_4_@CS-5 suspension in AMF with the intensity of 5.82 × 10^8^ Am^−1^s^−1^ for 10 min. The curves of temperature rise were monitored by using an optical fiber thermocouple (IF-C, Inno, Fuzhou, China) in real-time.

The specific loss power (SLP) of Fe_3_O_4_@CS nanoparticles was calculated by using Equation (1) [22].

(1) $$\mathrm{SLP}=\frac{CV_{s}}{m}\times \frac{dT}{dt}$$

where *C* is the volumetric heat capacity of solution, *Vs* is the sample volume, *m* is the mass of Fe_3_O_4_@CS nanoparticles, and *dT*/*dt* is the initial slope of the temperature increase curve with time. 

### 2.6. Hydrothermal Release of Fe_3_O_4_@CS-ACVA-TB Magnetic Nanoparticles

The Fe_3_O_4_@CS-ACVA-TB suspension (1 mg/mL) was incubated in water bath at 23, 37, 57, and 80 °C in PBS of pH 7.4. Then, 3 mL of supernatant was collected by centrifugal separation (8000 r/min) at the intervals of 0, 1, 3, 6, 9, 12, 24, 36, and 48 h and the absorbance value of supernatant at λ = 632 nm was detected by a UV spectrophotometer. Finally, the cumulative release rate of TB at different temperatures was calculated by Equation (2):

(2) $$\;\mathrm{cumulative}\;\mathrm{release}\;\%=\frac{\mathrm{drug}\;\mathrm{release}\;\mathrm{content}}{\mathrm{total}\;\mathrm{amount}\;\mathrm{of}\;\mathrm{drugs}}\times 100\%$$

### 2.7. A Single Magnetic Heat Control Release of Fe_3_O_4_@CS-ACVA-TB Magnetic Nanoparticles

Fe_3_O_4_@CS-ACVA-TB solution (1 mg/mL) was incubated at 23 °C in PBS of pH 7.4. Before being triggered by magnetic heating, 3 mL of supernatant was collected every 1 h by centrifugal separation (8000 r/min) and monitored for the absorbance value at λ = 632 nm with a UV spectrophotometer. After monitoring the release every 1 hour over the course of 4 h, the samples were exposed to an AMF with the intensity of 5.82 × 10^8^ Am^−1^s^−1^ for 5, 10, 15, and 20 min followed by recording the absorbance value every 1 h. The samples at 23 °C without AMF treatment performed as the blank control for this experiment.

### 2.8. Multiple Magnetic Heat Control Releases of Fe_3_O_4_@CS-ACVA-TB Magnetic Nanoparticles

Fe_3_O_4_@CS-ACVA-TB solution (1 mg/mL) was prepared and incubated at 23 °C in PBS of pH 7.4. Before being triggered by magnetic heating, 3 mL of supernatant was collected every 1 h by centrifugal separation (8000 r/min) and monitored for the absorbance value at λ = 632 nm with a UV spectrophotometer. After monitoring the release every 1 h over the course of 4 h, the samples were exposed to an AMF with the intensity of 5.82 × 10^8^ Am^−1^s^−1^ for 5 min, followed by monitoring the absorbance value of supernatant every 1 h. The next AMF was not applied until the release of TB leveled off. Three monitoring cycles were performed in total. Similarly, three cycles with AMF times of 10, 15, and 20 min were also performed. The samples at 23 °C without AMF treatment performed as the blank control for this experiment.

## 3. Results and Discussion

### 3.1. Synthesis and Characterization of Magnetic Fe_3_O_4_@CS Nanoparticles

For biomedical applications, chitosan, with rich active amino groups was chosen to wrap Fe_3_O_4_. The advantage of preparing chitosan-coated magnetic composites is that chitosan-conjugated particles can be easily prepared by using a co-precipitation method [23], because the chitosan’s amine groups (–NH_2_) with positive charge can easily interact with negatively charged groups (Fe–OH) on the magnetite nanoparticles (MNPs) through ion exchange reactions [16]. The prepared MNPs (Fe_3_O_4_@CS-NH_2_) allow for subsequent modification and drug loading. Then, to introduce excellent magneto-thermal response functionality, the azo compound ACVA was chosen as a thermal-sensitive switch, which is gradually being developed as a triggering agent for stimulation-responsive drug delivery systems [24,25]. Here, some of the carboxylic acid of ACVA firstly formed an amide bond with the prepared Fe_3_O_4_@CS-NH_2_ nanoparticles, and the product was named as Fe_3_O_4_@CS-ACVA. Subsequently, the remaining carboxylic acid of ACVA formed another amide bond with the amine groups on TB, and the final particles were named Fe_3_O_4_@CS-ACVA-TB.

**Figure 1:**
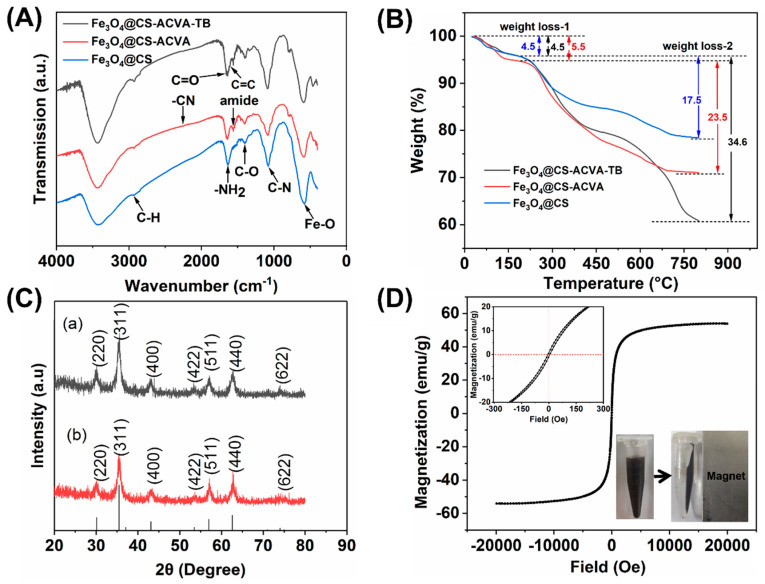
(**A**) Infrared spectroscopy and (**B**) thermogravimetric analysis of Fe_3_O_4_@CS-ACVA-TB, Fe_3_O_4_@CS-ACVA, and Fe_3_O_4_@CS, respectively. (**C**) XRD pattern of (a) Fe_3_O_4_@CS and (b) Fe_3_O_4_@CS-ACVA-TB. (**D**) Magnetization curve of Fe_3_O_4_@CS, nanoparticles at 300 K. The inset shows the magnetization curves in a small scale and the magnet attracting the Fe_3_O_4_@CS nanoparticles.

The products of each step of the grafting process were analyzed by infrared spectroscopy. As shown in Figure 1A, the characteristic peaks at 1626 cm^−1^ correspond to the N-H stretching vibration in chitosan, 1392 cm^−1^ corresponds to the C-O bending vibration in chitosan, 2922 cm^−1^ corresponds to the C-H stretching vibration in chitosan, and 1039 cm^−1^ corresponds to the C-N vibration in chitosan. A new characteristic absorption peak formed at 1562 cm^−1^ corresponds to the amide bond, which confirms that ACVA is successfully connected via the amide bond surface of Fe_3_O_4_@CS magnetic nanoparticles. In addition, the C≡N absorption at 2253cm^−1^, the absorption peaks at 1719 cm^−1^ (C=O stretching), 1451 cm^−1^ (C-H bending) and 2904 cm^−1^, 2934 cm^−1^ and 2981 cm^−1^ (C-H stretching) also prove that ACVA was successfully grafted on Fe_3_O_4_@CS. Furthermore, the absorption at 1612 cm^−1^ corresponds to the stretching vibration of C=C of the benzene ring on TB, which indicates that the model drug molecule was successfully grafted onto the surface of Fe_3_O_4_@CS nanoparticles through the ACVA.

TGA was used to analyze the weight of organic magnetic nanoparticles (Figure 1B). As crystalline water was removed (25~200 °C), the weight loss rate of Fe_3_O_4_@CS, Fe_3_O_4_@CS-ACVA and Fe_3_O_4_@CS-ACVA-TB was 4.5 %,5.5 % and 4.5 %, respectively. The mass of the three samples decreased 17.5 %, 23.5 % and 34.6 %, respectively, after continuing to heat from 200~800 °C under N_2_ protection. The results showed that ACVA and TB were present on the nanoparticles’ surface in weights of 6.4% and 11.1%, respectively.

Subsequently, Figure 1C shows the XRD patterns of Fe_3_O_4_@CS (a) and Fe_3_O_4_@CS-ACVA-TB (b). The diffraction peaks observed at 30.06°, 35.41°, 43.03°, 53.39°, 56.91°, 62.49°, and 73.93° correspond, respectively, to (220), (311), (400), (442), (511), (440) and (622) sharp crystal structure planes of Fe_3_O_4_ (JCPDS 76-1849), which illustrated that the later grafting process did not change the Fe_3_O_4_ nanoparticles. The average crystallite size of the chitosan-coated Fe_3_O_4_ nanoparticles was calculated using the standard Debye–Scherer equation.

(3) $$\;D=\frac{k\lambda }{\beta \cos\theta }$$

where, D is the crystallite particle size in nm, k refers to grain shape factor taken as unity (based on assumption that shape of particles is spherical), λ is the incident X-ray wavelength of Cu-Kα source of radiation, and θ is the Bragg’s angle, while β (in radians) refers to broadening of diffraction line measured full width at half maximum intensity (FWHM). The average crystallite size of Fe_3_O_4_@CS and Fe_3_O_4_@CS-ACVA-TB nanoparticles was 8.97 and 7.94 nm, respectively. The magnetic properties of the Fe_3_O_4_@CS-1 were characterized by a vibrational sample magnetometer. Figure 1D shows the saturation magnetization (*M_s_*) of Fe_3_O_4_@CS-1 nanoparticles synthesized was 54.0 emu/g at 300 K, which is lower than the saturation magnetization of bare Fe_3_O_4_ reported in the previous literature (80–100 emu/g) [26]. The reason for the reduced saturation magnetization of the sample might come from the non-magnetic component chitosan of the nanocomposites. Moreover, the value of *M_r_*/*M_s_* < 0.1 (in Table 1) indicates that the synthesized nanoparticles were superparamagnetic, which is a propitious character of magnetic nanocarriers such as DDS [27].

**Figure 2:**
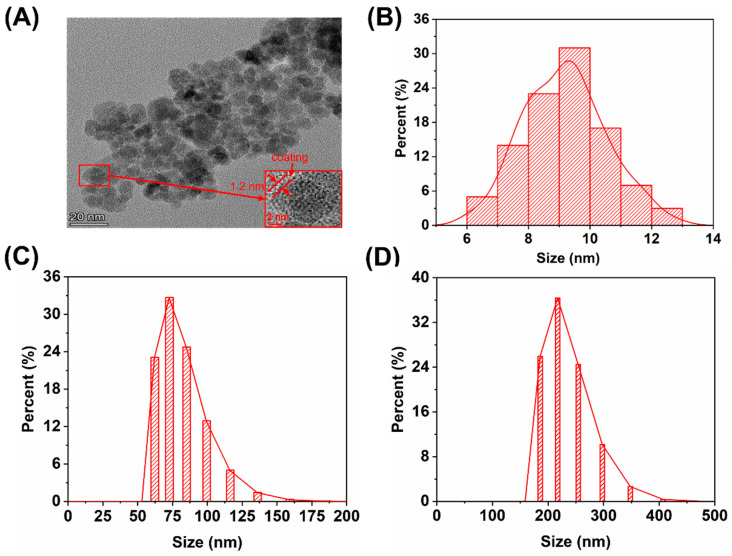
(**A**)Transmission electron micrographs and (**B**) particle size distribution maps of Fe_3_O_4_ @CS-ACVA-TB. The hydrated particle size distribution maps of (**C**) Fe_3_O_4_@CS and (**D**) Fe_3_O_4_@CS-ACVA-TB are from DLS.

Figure 2A shows the TEM image of Fe_3_O_4_@CS-ACVA-TB, which was found to be spherical particles with an average particle size of 9.2 ± 1.3 nm (Figure 2B). As Figure 2A shows, the prepared particles exhibit a certain degree of aggregation, which may be caused by strong interactions between chitosan molecules. We analyzed the distance between particles in the TEM image, which was performed by measuring 20 pairs of nanoparticles in the adjacent area of the image to estimate the coating thickness of Fe_3_O_4_ particles to be about 1.2 nm.

Additionally, through the dynamic light scattering scanner, the average hydrated particle size of Fe_3_O_4_@CS and Fe_3_O_4_@CS-ACVA-TB (Figure 2C,D) was found to be 80.0 ± 16.6 and 230.6 ± 40.6 nm, respectively. This size is much larger than that charactered by TEM. On the one hand, the hydrated particle size of nanoparticles would increase with the increase in the surface modifier during the material synthesis. On the other hand, this might be due to the association and aggregation of Fe_3_O_4_ nanoparticles in water through the action of van der Waals’ forces and/or hydrogen bonds. 

### 3.2. The Magneto-Thermal Properties of Fe_3_O_4_@CS Magnetic Nanoparticles

**Figure 3:**
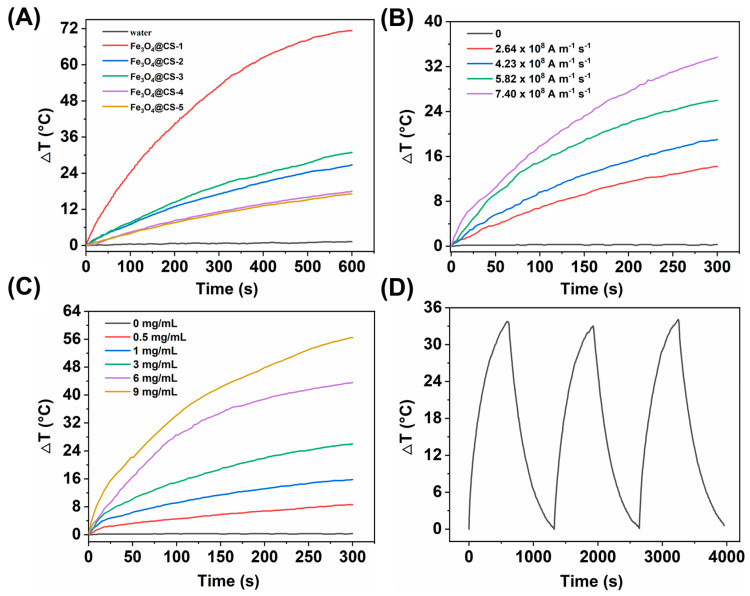
Magneto-thermal curves of Fe_3_O_4_@CS nanoparticles with (**A**) different feeding ratios of chitosan, (**B**) different intensities of AMF, and (**C**) different concentrations of Fe_3_O_4_@CS. (**D**) Magneto-thermal cycle stability of Fe_3_O_4_@CS.

To investigate the magneto-thermal conversion capability of Fe_3_O_4_@CS nanoparticles exposed to AMF, we changed some possible conditions, including AMF intensity (the safety condition *H*_0_ *f* < 5 × 10^9^ Am^−1^s^−1^) [28], the concentration of MNPs, and the content of chitosan of MNPs. Due to the Néel–Brown mechanism, the magnetic component in the nanoparticles was heated when exposed to AMF, causing a temperature increase. However, the magneto-thermal response ability of Fe_3_O_4_@CS gradually decreased with the increase in non-magnetic components (Figure 3A). The corresponding SLP values were 1162.6, 266.7, 209.2, 118.4, and 93.0 W/g (in water). Therefore, Fe_3_O_4_@CS-1 magnetic nanoparticles were selected for further experiments and abbreviated as Fe_3_O_4_@CS.

Figure 3B shows the heating situation of the Fe_3_O_4_@CS suspension (3 mg/mL) exposed to different AMF intensities. With the increase in the applied AMF intensity, the sample temperature increased by 14.1, 19.1, 26.1, and 33.8 °C from 26.1 °C within 5 min, indicating that the prepared Fe_3_O_4_@CS nanoparticles have good magneto-thermal properties. Figure 3C depicts the temperature-rising curve within 5 min of concentrations of 0.5, 1, 3, 6, and 9 mg/mL Fe_3_O_4_@CS suspensions exposed to the fixed AMF (5.82 × 10^8^ Am^−1^s^−1^). Moreover, the temperature increased 8.7, 15.9, 26.1, 43.7 and 56.6 °C from 26.1 °C within 5 min, indicating that the heating rate accelerates with the increase in concentration of Fe_3_O_4_@CS. The results of the three magneto-heat-cooling cycles (Figure 3D) show that the maximum temperature, which can be reached by the Fe_3_O_4_@CS nanoparticles, is almost unchanged when exposed to AMF for the same conditions, which indicates that it possesses good magneto-thermal cycle stability and can be subjected to multiple magnetic heating operations. Nevertheless, the tolerable temperature of normal tissue or cells is about 43 °C [29]. The concentration of 1 mg/mL of Fe_3_O_4_@CS nanoparticles and the AMF with the intensity of 5.82 × 10^8^ Am^−1^s^−1^ was selected to further experiments to avoid damage to the tissue or cells caused by excessive temperature and to better evaluate the performance of the drug delivery model.

### 3.3. In Vitro Release Study

#### 3.3.1. Hydrothermal Release of Fe_3_O_4_@CS-ACVA-TB Magnetic Nanoparticles

**Figure 4:**
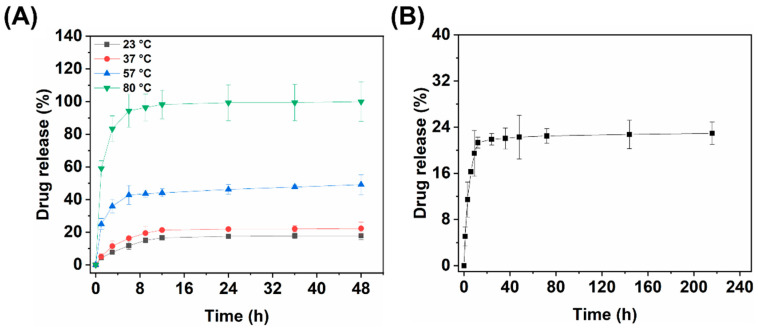
Release of Fe_3_O_4_@CS-ACVA-TB: (**A**) the release efficiency of TB from Fe_3_O_4_@CS nanoparticles in water bath at 23, 37, 57 and 80 °C. (**B**) the long-term release of TB at 37 °C. (n = 3).

To investigate the temperature sensitivity performance of ACVA-modified Fe_3_O_4_@CS nanocarriers, the TB with high molar absorption coefficient, outstanding thermal stability, and water solubility was used as a model drug molecule. It has been reported that the half-life of the azo compound is 10 h at 57 °C, while the azo chemical bond is nearly broken completely at 80 °C after 48 h [30]. Therefore, the effects of temperature on TB release from nanocarriers were investigated at four different water bath temperatures, 23, 37, 57 and 80 °C, in PBS of pH 7.4, and the cumulative release of TB after 48 h of incubation at 80 °C was used as the total amount of drug. As shown in Figure 4A, higher temperatures show faster and more drug release. The cumulative release of TB finally reached 17.8 ± 2.5%, 22.3 ± 3.8%, and 49.1 ± 6.1% at 23, 37 and 57 °C after 48 h, respectively. Furthermore, Figure 4 (A) shows the release behavior of Fe_3_O_4_@CS-ACVA-TB nanoparticles with two phases, a rapid release within 9 h, and followed from 9 to 48 h, a performance of plateau period (barely released at 23 and 37 °C) or slow release (57 °C). The rapid release during the inaugural 9 h is likely to involve a diffusion mechanism of the dye loaded on the chitosan coating [31]. The plateau period (23 and 37 °C) that occurred from 9 to 48 h proved that most of the dye was immobilized within the Fe_3_O_4_@CS nanocarriers due to the molecular bonding of ACVA. When the temperature continued to increase (57 °C), the azo bond in ACVA became unstable at this time, resulting in the slow release of TB immobilized by ACVA as well. To investigate the long-term stability of the material in the solution, we continued to monitor the concentration of dye in the Fe_3_O_4_@CS-ACVA-TB suspension (1 mg/mL), which had been incubated at 37 °C in PBS of pH 7.4 for 48 h. As shown in Figure 4B, the concentration of TB in the centrifuge tube remained almost unchanged when the time was prolonged to the 9th day. This indicated that the drug delivery model prepared with Fe_3_O_4_@CS as the magnetic core and ACVA as the connecting bridge had good stability, which lays a foundation for future application.

#### 3.3.2. Kinetic-Analytic Studies of Drug Release

To explain the release behavior of Fe_3_O_4_@CS-ACVA-TB at different temperatures, different equations, such as zero-order, first-order, Higuchi and Korsemeyer–Peppas, were used for analysis [32]. Equations (3)–(6), corresponding to each model, are shown below:

(i) Zero-order:
  (4) $$\frac{M_{t}}{M_{\infty }}=kt$$
(ii) First-order:
  (5) $$ln\left(1-\frac{M_{t}}{M_{\infty }}\right)=-kt$$
(iii) Higuchi:
  (6) $$\frac{M_{t}}{M_{\infty }}=kt^{\frac{1}{2}}$$
(iv) Korsemeyer–Peppas:
  (7) $$\frac{M_{t}}{M_{\infty }}=kt^{n}\;$$
  where *M_t_* is the total amount of TB released at time *t*, *M*_∞_ is the total amount of drug released as time goes to infinity (i.e., complete release), *k* is a release constant, and *n* is the release exponent that is used to characterize different release mechanisms. For spherical particles, the value of *n* indicates Fickian diffusion if *n* < 0.5, nonFickian or anomalous phenomena if 0.5 < *n* < 1, and *n* > 1 implies a lack of time dependence on release kinetics (i.e., zero-order kinetics) [33].

**Figure 5:**
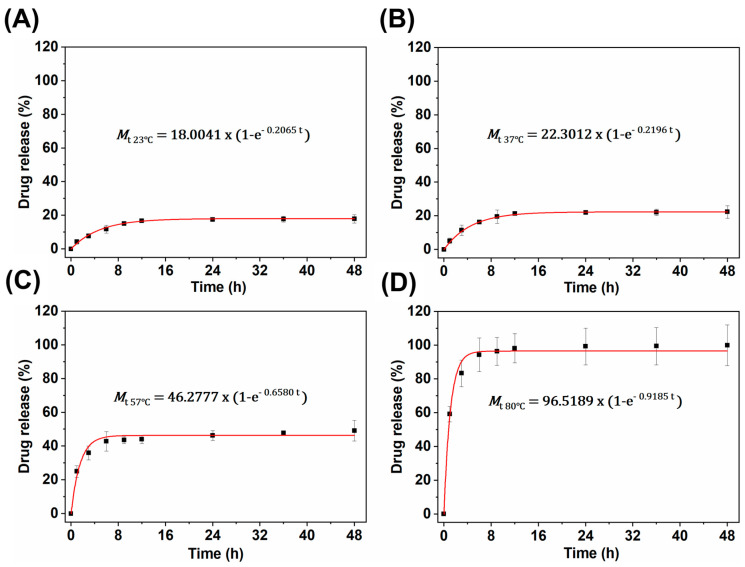
First-order model fitting curves and their equations for (**A**) 23 °C, (**B**) 37 °C, (**C**) 57 °C and (**D**) 80 °C.

The release constants and correlation coefficients corresponding to each model are shown in the Table 2, which with a high “R^2^” value was considered as the best-fitting model. All values of the R^2^ of the first-order release model for nanoparticles were higher than the zero-order model at different temperatures, indicating that the leakage of drugs from nanoparticles follows first-order kinetics. This is because the solubility of polymer chitosan in PBS of pH 7.4 is approximately low, so that the water content in the shell is approximately low, which would induce the low permeability of TB [21]. Therefore, when the thermal decomposition of ACVA occurred continuously in nanoparticles, it would lead to a higher concentration of TB inside the chitosan coating than that in the external solution, thus showing a concentration-dependent diffusion behavior. Figure 5A–D shows the First-order fitted curves at 23, 37, 57 and 80 °C, respectively. All the “*n*” values of the Korsmeyer–Peppas equation for nanoparticles were less than 0.5, indicating that the drug release followed Fickian diffusion. Moreover, the release constants *k* in the First-order and Korsmeyer–Peppas models increased with the increase in temperature, indicating that the increase in temperature accelerates the breakage rate of ACVA and the thermal motion of molecules in solution, leading to a rapid increase in drug concentration in the nanoparticles. This is as expected and provides us with the possibility to subsequently investigate the magneto-thermal release behavior of nanoparticles.

### 3.4. Magneto-thermal Controlled Release of Fe_3_O_4_@CS-ACVA-TB Nanoparticles

Here, we used two ways of applying AMF to investigate the effect of AMF on the release of TB in Fe_3_O_4_@CS-ACVA-TB: (a) single exposures for a different time and (b) multiple sequential exposures each for the same time.

#### 3.4.1. Single Magneto-thermal Controlled Release of Fe_3_O_4_@CS-ACVA-TB Nanoparticles

**Figure 6:**
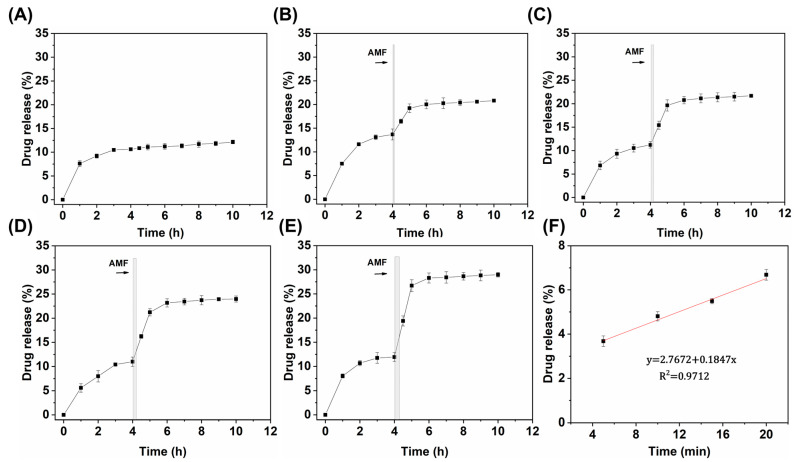
Magnet-controlled drug release plots of Fe_3_O_4_@CS-ACVA-TB nanoparticles: exposed to AMF for (**A**) 0 min, (**B**) 5 min, (**C**) 10 min, (**D**) 15 min and (**E**) 20 min. (**F**) The fitting curve of TB release content after different action times of AMF. (n = 3).

To confirm the robustness of the ACVA, Fe_3_O_4_@CS-ACVA-TB suspension (1 mg/mL) was placed at 23 °C before being exposed to AMF. After monitoring the release of dye every 1 h over the course of 4 h with a UV spectrophotometer, the samples were exposed to an AMF (5.82 × 10^8^ Am^−1^s^−1^) for 5, 10, 15, and 20 min. As shown in Figure 6A–E, about 12.5% release of dye was detected in the first 4 h at 23 °C before exposure to an AMF, showing that the tightness of the ACVA can prevent early leakage of most parts of the TB in nanoparticles. After being exposed to AMF for different times, similar release profiles of TB were observed in Figure 6B–F: (i) after AMF, the burst increase in release for 1 h; (ii) after 1 h, the release efficiency of TB slowed down as the magnetic nanoparticles returned to the original temperature. Moreover, the concentration of TB increased 3.7 ± 0.2%, 4.8 ± 0.2%, 5.5 ± 0.1%, and 6.7 ± 0.2% after 5, 10, 15, and 20 min of AMF trigger time, respectively. The results of the linear fit of the increments of TB concentrations controlled by AMF suggested that the release of the drug was correlated with the AMF trigger time (Figure 6F).

#### 3.4.2. Multiple Magneto-thermal Controlled Release of Fe_3_O_4_@CS-ACVA-TB Nanoparticles

**Figure 7:**
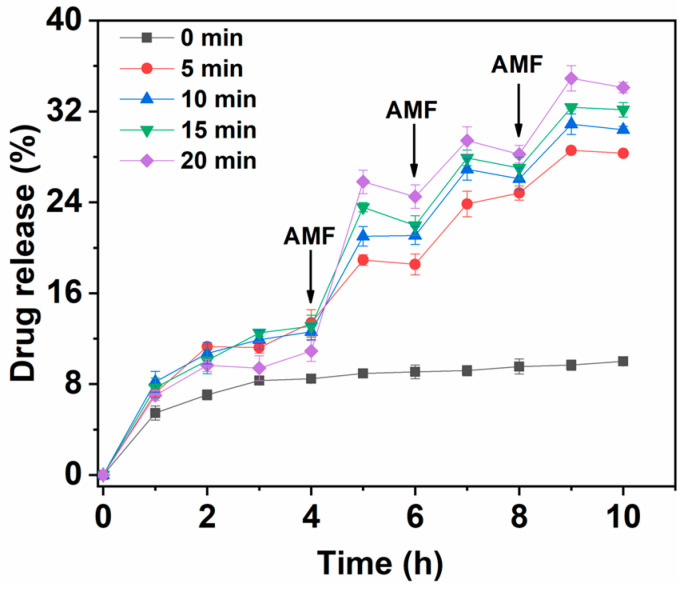
Time-dependent release profile of TB from Fe_3_O_4_@CS-ACVA-TB through magnetic actuation under AMF for 3 cycles. (n = 3).

The second type of release study was performed with multiple sequential exposures of AMF to explore whether the material could achieve the effect of multiple controlled releases. To confirm the robustness of the ACVA, Fe_3_O_4_@CS-ACVA-TB suspension (1 mg/mL) was placed at 23 °C and was monitored for the release of dye every 1 h. The leakage of dye was less than 15%, observed in the first 4 h at 23 °C before exposure to the first cycle of AMF triggering (5.82 × 10^8^ Am^−1^s^−1^). A total of three cycles of AMF triggering were performed at room temperature, and the AMF triggering time was 5, 10, 15, and 20 min in every cycle, respectively. The release efficiency of the drug increased with the increase in the AMF time during each magneto-thermal cycle (Figure 7), which is similar with that described above for a single application of AMF. Subsequently, when the applied AMF was withdrawn, the drug release efficiency showed a subsequent increase followed by a plateau. This release pattern reappeared when both the second and third magnetic fields were applied.

As above results showed, it is evident that Fe_3_O_4_@CS nanocomposites have an excellent ability to control drug release by magnetic heating. When AMF is applied, the magnetic nanoparticles would respond rapidly to generate local high temperature, which will break the azo bond in ACVA and then affect the release kinetics of the drug from the nanoparticles, accelerating the release of the drug. With the withdrawal of AMF, the nanoparticles would gradually stabilize, and the release behavior of the drug therein would again follow the release kinetics at the ambient temperature that they are exposed to. Moreover, multiple magneto-thermal releases of Fe_3_O_4_@CS nanocomposites can be achieved by controlling the switch of the AMF generation device as well.

## 4. Conclusions

In summary, CS-modified magnetic nanoparticles (Fe_3_O_4_@CS), with a high magnetization (54.0 emu/g), were successfully synthesized by the co-precipitation method. Subsequently, the temperature-sensitive molecule ACVA and the amino-based model drug toluidine blue were modified on the surface of Fe_3_O_4_@CS and were used to illustrate that the nanocarriers could control drug release. In vitro hydrothermal release studies showed that in the absence of AMF, most parts of the TB would be effectively enclosed within the nanocarriers at lower ambient temperatures (23 or 37 °C) due to the molecular bonding of ACVA, and the ACVA could remain unbroken for a longer period (at least 9 days in 37 °C). However, with the increase in incubation temperature (57 °C), the ACVA would become unstable, resulting in the slow release of TB immobilized by ACVA. Moreover, the results of kinetic fitting of hydrothermal release data showed that TB released from nanoparticles followed first-order kinetics (R^2^ > 0.99) and the Korsemeyer–Peppas model (R^2^ > 0.99, n < 0.5). In the next experiments, when the Fe_3_O_4_@CS-ACVA-TB nanoparticles were exposed to the AMF, the magnetic core would respond rapidly to generate local high temperature, which would break the azo bond in ACVA and then affect the release kinetics of the drug from the nanoparticles. We effectively controlled the release of TB by adjusting the time of applied AMF on the magnetic nanoparticles, and the results showed an approximately linear relationship between the amount of drug release and the time of AMF action (R^2^ = 0.9712). Moreover, the drug could be released gradually even after several magneto-thermal cycles. These results indicated that the ACVA-modified Fe_3_O_4_@CS nanocarriers as a magneto thermally responsive drug-controlled release model processes a significant potential in controlling the temperature and drug dose.

## Figures and Tables

**Table 1:** Magnetic properties of Fe_3_O_4_@CS-1. *M_s_* is the saturation magnetization, and *M_r_* is the remanence magnetization.

Sample	*M_s_* (emu/g)	*M_r_* (emu/g)	*M_r_*/*M_s_*
Fe_3_O_4_@CS-1	54.0057	0.1395	0.0026

**Table 2:** The drug release kinetic parameters for synthesized Fe_3_O_4_@CS-ACVA-TB nanoparticles.

The Drug Release Kinetics	Constants	Correlation Coefficients
Zero-order release kinetics	*k*_23 °C_ = 1.1256	R^2^_23 °C_ = 0.8499
	*k*_37 °C_ = 1.4637	R^2^_37 °C_ = 0.6784
	*k*_57 °C_ = 1.4679	R^2^_57 °C_ = 0.7966
	*k*_80 °C_ = 3.5318	R^2^_80 °C_ = 0.4294
First-order release kinetics	*k*_23 °C_ = 0.2065	R^2^_23 °C_ = 0.9997
	*k*_37 °C_ = 0.2196	R^2^_37 °C_ = 0.9998
	*k*_57 °C_ = 0.6580	R^2^_57 °C_ = 0.9978
	*k*_80 °C_ = 0.9185	R^2^_80 °C_ = 0.9985
Higuchi model	*k*_23 °C_ = 4.5645	R^2^_23 °C_ = 0.9787
	*k*_37 °C_ = 5.9771	R^2^_37 °C_ = 0.9628
	*k*_57 °C_ = 8.8708	R^2^_57 °C_ = 0.9275
	*k*_80 °C_ = 23.5588	R^2^_80 °C_ = 0.7873
Korsmeyer–Peppas model	*k*_23 °C_ = 8.9168, *n*_23 °C_ = 0.2378	R^2^_23 °C_ = 0.9946
	*k*_37 °C_ = 10.6153, *n*_37 °C_ = 0.2370	R^2^_37 °C_ = 0.9958
	*k*_57 °C_ = 31.2571, *n*_57 °C_ = 0.1223	R^2^_57 °C_ = 0.9975
	*k*_80 °C_ = 64.7859, *n*_80 °C_ = 0.1415	R^2^_80 °C_ = 0.9912

## Data Availability

The data that support the findings of this study are available from the corresponding author upon reasonable request. All figures in this paper are original.
